# From waste to strength: The role of FGD gypsum in loess stabilization and its environmental benefits

**DOI:** 10.1016/j.isci.2025.113909

**Published:** 2025-10-30

**Authors:** Yuan Kangze, Xie Wanli, Gao Xuanyu, Li Xinyu, Liu Qiqi

**Affiliations:** 1 Department of Geology, Northwest University, Xi’an, Shaanxi, China; 2 China-Kyrgyzstan Belt and Road Joint Laboratory on Special Geotechnical Dynamic Disaster Prevention and Control, Northwest University, Xi’an, Shaanxi, China; 3 Xi’an Key Laboratory of Prevention of Loess Dynamic Disaster and Restoration of Environment, Northwest University, Xi’an, Shaanxi, China

**Keywords:** Environmental science, Applied sciences, Materials science

## Abstract

Loess exhibits high porosity and structural vulnerability, presenting serious geotechnical challenges in arid and semi-arid regions. In this study, the use of flue gas desulfurization (FGD) gypsum, a coal combustion by-product, as a sustainable alternative to traditional stabilizers was investigated. The resulting mechanical properties showed that incorporating 20% FGD gypsum could increase the unconfined compression strength of loess by about four times. The microstructural characterization (FTIR, XRD, SEM, and CT) confirmed improved cementation via CaSO_4_·2H_2_O formation and a denser microstructure with higher microporosity. Avizo-based image processing further elucidated the micro-macro correlations. Life cycle assessment was also carried out, which showed up to 97% lower global warming potential than lime. These results demonstrate that FGD gypsum significantly enhances mechanical performance and environmental sustainability, offering a viable low-carbon alternative for loess stabilization.

## Highlights

• FGD gypsum effectively reduces loess porosity and increases UCS performance
• Microstructure evolution was revealed by SEM, EDX, and 3D CT image analysis
• Pore network modeling confirms the gypsum-induced densification of loess structure
• Multi-scale testing reveals stabilization mechanisms at the structural and pore level

## Graphical abstract

**Figure:**
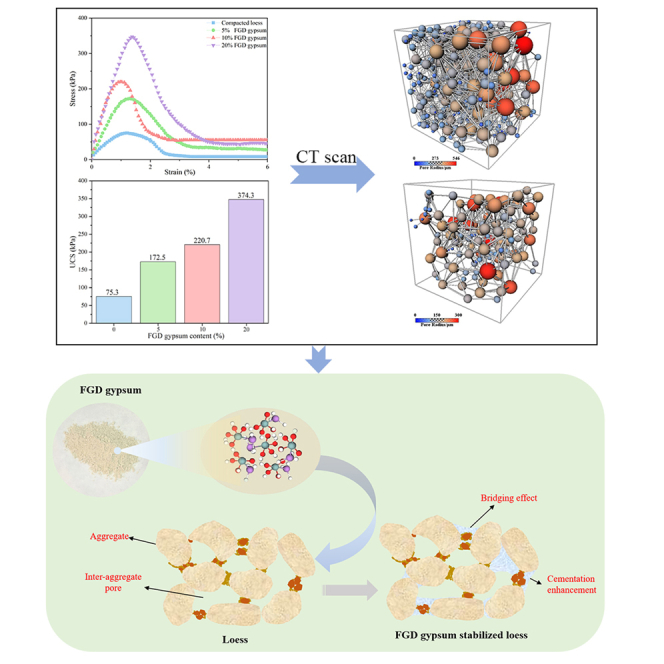

## Introduction

Loess is a critical soil type, covering approximately 10% of the Earth’s surface, and is predominantly found in arid and semi-arid regions.^1^ It has high porosity, loosely packed particle arrangement, and a meta-stable structure, exhibiting pronounced vulnerability to external disturbances and making it highly prone to erosion, structural collapse, and significant deformation when subjected to environmental forces or human activities.^2^^,^^3^ These characteristics not only raise soil management issues but also negatively impact agricultural productivity and environmental stability, especially in regions such as the Loess Plateau, where large-scale agricultural and infrastructure projects are undertaken.^4^ Thus, improving its properties is critical. In recent years, numerous alternative techniques have been proposed to improve the engineering performance of loess, including bio-cementation through microbial-induced calcium carbonate precipitation (MICP),^5^^,^^6^ enzymatic stabilization, and foundation optimization under coupled loading conditions,^7^ which have demonstrated promising results in improving loess structure, shear resistance, and collapsibility. However, their practical application is often limited by their high cost, environmental sensitivity, and large-scale adaptability.

China’s continued dependence on coal as a primary energy source, accounting for about half of global consumption^8^ has increased environmental concerns, particularly the release of sulfur dioxide (SO_2_), a major air pollutant.^9^^,^^10^ In order to limit such emissions, implementing flue gas desulfurization (FGD) systems is mandated, resulting in the large-scale generation of FGD gypsum, a calcium-rich industrial by-product. Although FGD gypsum is commonly used in cement and gypsum-based construction products,^9^ the persistently increasing stockpiles of FGD gypsum necessitate alternative, sustainable, and high-volume reuse pathways.

Considering the potential of FGD gypsum, its utilization as an alternative stabilizer for loess has environmental and engineering advantages. The chemical composition of FGD gypsum facilitates hydration reactions that form crystalline compounds, such as ettringite and gypsum, effectively filling pores, enhancing inter-particle bonding, and improving overall soil strength and water resistance.^10^ Its moderate expansive behavior during hydration can also mitigate shrinkage-induced cracking, improving dimensional stability under drying-wetting cycles.^11^ Compared to traditional stabilizers, such as lime and cement, FGD gypsum has several distinctive benefits. While lime-stabilized soils are vulnerable to carbonation and sulfate attack, and cement-treated soils are often sensitive to environmental variations that degrade mechanical performance,^12^^,^^13^ FGD gypsum exhibits better long-term chemical stability and durability. Also, unlike lime and cement, whose production processes are highly energy-intensive with significant release of carbon emisisons,^14^^,^^15^ FGD gypsum is an industrial by-product with a substantially lower carbon footprint, aligning with current environmental sustainability and resource recovery goals.^16^ Nevertheless, using FGD gypsum for loess stabilization has remained unaddressed. The previous research studies have focused on its application in cementitious composites or agriculture, while the systematic evaluation of its performance in loess, particularly for strength improvement, microstructure evolution, and environmental implications, is still lacking. Therefore, exploring the use of FGD gypsum provides a promising low-carbon alternative to conventional binders and fills a critical research gap in sustainable soil stabilization practices.

Macroscopic characteristics are largely determined by their microstructure, as the correlation is widely demonstrated in various studies.^17^^,^^18^^,^^19^ The research on the interactions between microstructure and macroscopic behavior, including UCS,^20^ shear behavior,^21^ and water retention capacity,^22^ has also been established. Due to its fine particle size and large specific surface area, adding FGD gypsum to loess may alter its microstructure, improving mechanical behavior and environmental performance. Therefore, comprehensive microstructural observations are essential to understanding the mechanisms of loess stabilization with FGD gypsum. Among different microstructural characterization techniques, scanning electron microscopy (SEM) is quite popular and widely used because of easier sample preparation, exceptional resolution, and extensive applicability across various materials.^23^^,^^24^ However, SEM yields qualitative imaging data, limiting its utility in detailed scientific analysis. Thus, for quantitative microstructural parameters, particularly for loess, advanced image processing of SEM imaging is crucial. In this context, Avizo software has emerged as a preferred tool, enabling rigorous analysis and extraction of critical microstructural metrics.^25^^,^^26^ Developed by Thermo Fisher Scientific, Avizo is an advanced 3D visualization and analysis tool that efficiently processes images, accurately identifying and segmenting microstructural elements such as particles and pores in loess samples. While SEM results offer 2D structural parameters, which capture only part of the pore characterization, especially for irregular or elongated pores, combining SEM with computed tomography (CT) to establish 3D structural models allows for a more complete interpretation of the mechanisms underlying loess stabilization with FGD gypsum. Despite the potential for multidimensional analysis, research in this area remains limited.

Whereas recent studies have explored the environmental impact of FGD gypsum application in soils,^27^ the findings on the detailed environmental implications of the stabilization process are sparse. In order to fill this research gap, life cycle assessment (LCA), a promising tool for evaluating the environmental impact of the entire process, including transportation, production, application, and waste disposal, with wide potential applications,^28^^,^^29^^,^^30^ was adopted in this study. LCA can help identify key environmental impact categories and contributors within the system, facilitating decision-making to mitigate negative environmental effects.

In this study, the mechanical properties of loess stabilized with varying proportions of FGD gypsum were systematically evaluated. Also, the comprehensive microstructural analysis was conducted to correlate with cementation strength, mineralogical composition, morphological characteristics, and pore structure. Further, the environmental implications of the stabilization process were critically assessed. The potential of FGD gypsum as an effective and sustainable stabilizer for loess, contributing to both mechanical reinforcement and environmental sustainability, was elucidated. By integrating UCS, SEM, CT, and quantitative 2D and 3D image analyses using Avizo software, novel insights into the micro-macro mechanisms of loess stabilization were explored. Additionally, LCA provides a comprehensive evaluation of the environmental implications of LCS, highlighting the benefits of reusing industrial by-products in low-carbon geotechnical applications, which were evaluated. These findings help understand the underlying mechanisms of FGD gypsum-stabilized loess, bridging performance enhancement with environmental responsibility.

## Results and discussion

### Unconfined compression strength

**Figure 1:**
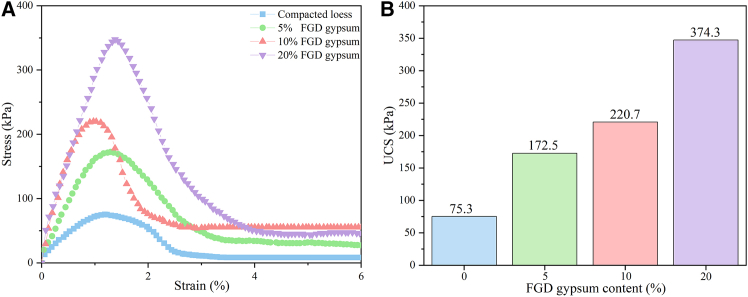
Results of unconfined compression strength (UCS) tests (A)Stress-strain curves and (B)unconfined compression strength.

The UCS of loess was significantly enhanced by adding FGD gypsum, as shown in Figures 1A and 1B. Figure 1A shows the stress-strain curves, indicating a progressive increase in peak strength with higher FGD gypsum content. Compacted loess (without FGD gypsum) exhibited the lowest UCS at 75.3 kPa, whereas incorporating 5%, 10%, and 20% FGD gypsum increased the UCS to 172.5 kPa, 220.7 kPa, and 374.3 kPa, respectively. These values are summarized in Figure 1B, highlighting the positive correlation between FGD gypsum dosage and strength development. Figure 1A also shows that all specimens exhibited brittle failure, evidenced by a sharp stress drop after reaching the peak. The 20% FGD gypsum sample, while achieving the highest peak stress, showed the steepest post-peak decline, indicating increased brittleness at higher dosages. This observation suggests that although FGD gypsum improves UCS, it may reduce ductility at high dosages, which should be considered in engineering applications. Overall, the results confirm that FGD gypsum is a promising stabilizer for loess, effectively enhancing its mechanical performance within the tested range of 0–20% content.

### Fourier transform infrared

**Figure 2:**
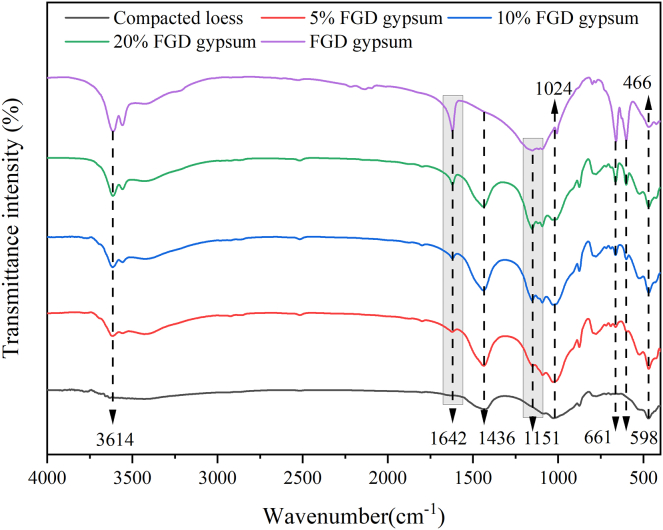
FTIR spectra of samples with different FGD gypsum

FTIR spectroscopy is an effective tool for identifying functional groups adsorbed on the surface of sample particles.^31^ Figure 2 shows the transmittance intensity for various samples, with different wavenumbers corresponding to specific functional groups. The strength of the functional group is inversely proportional to the transmittance intensity. For FGD gypsum, absorption peaks around 3614 cm^−1^ and 1624 cm^−1^ are attributed to hydroxyl groups (–OH), which likely originate from the crystalline water present in the hydrated form of the gypsum. A strong and broad peak at 1155 cm^−1^ indicates the sulfate group (–SO_4_), while additional peaks at 661 cm^−1^ and 598 cm^−1^ further confirm the presence of sulfate groups.^32^ The FTIR spectrum of compacted loess reveals four prominent absorption bands at 3614 cm^−1^, 1436 cm^−1^, 1024 cm^−1^, and 466 cm^−1^. The peaks at 3614 cm^−1^ are linked to the stretching vibrations of surface hydroxyl groups on the loess particles, while the band at 1436 cm^−1^ is associated with the bending vibrations of carbonate groups (–CO_3_). The broad peak at 1024 cm^−1^ corresponds to the antisymmetric stretching vibration of Si–O–Si, and the sharp peaks at 466 cm^−1^ are attributed to the symmetric stretching vibrations of Si–O–Si, indicating silicate anions (SiO_4_^2−^) on the surface of the loess particles. These findings confirm the existence of hydroxyl, carbonate, and silicate anions on the surface of the loess microspheres. Upon the addition of FGD gypsum, the FTIR spectra of the samples exhibit notable changes, particularly with the emergence of new peaks at 1624 cm^−1^, 1151 cm^−1^, 661 cm^−1^, and 598 cm^−1^, corresponding to hydroxyl and sulfate functional groups, respectively. This shift is due to the incorporation of CaSO_4_·2H_2_O from the FGD gypsum, which introduces a substantial amount of –OH and –SO_4_ groups into the loess structure. As the FGD gypsum content increases, the intensity of these functional groups strengthens, improving the mechanical properties by enhancing structural integrity.

### Mineralogy

**Figure 3:**
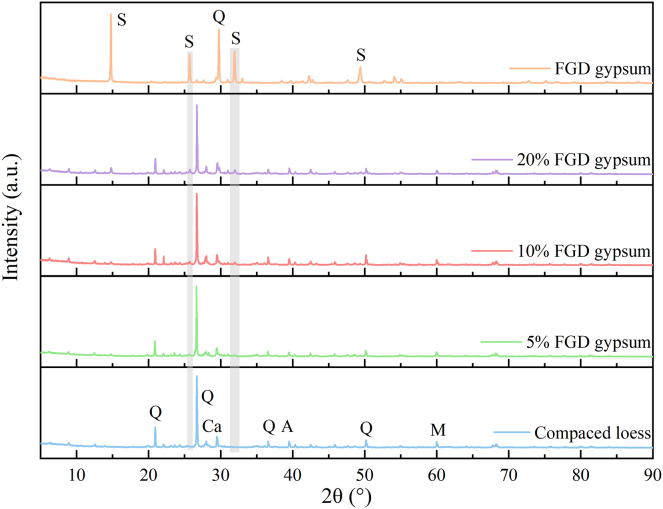
XRD patterns of samples (quartz (Q), calcite (Ca), albite (A), muscovite (M), and calcium sulfate dihydrate gypsum (S))

Figure 3 presents the XRD patterns results for FGD gypsum and loess with varying FGD gypsum contents. The horizontal axis represents the 2θ angle, and the short vertical ticks are interval markers spaced every 5°, included to improve scale readability. For the FGD gypsum, distinct diffraction peaks are observed at 14.8°, 25.7°, 31.9°, and 49.41°, corresponding to calcium sulfate dihydrate (CaSO_4_·2H_2_O), consistent with previous findings.^33^ Additionally, a characteristic quartz (SiO_2_) peak is detected at 29.7°. For compacted loess, diffraction peaks within the 5°–90° 2θ range indicate non-clay minerals, such as quartz (SiO_2_), albite (Na(AlSi_3_O_8_)), and calcite (CaCO_3_), along with muscovite (K_0_._77_Al_1_._93_(Al_0_._5_,Si_3_._5_)O_10_(OH)_2_) as the primary clay mineral (Ma et al., 2017). FGD gypsum addition to loess alters its mineralogical composition, as validated by XRD. The 5% FGD gypsum sample exhibits new peaks at 14.8° and 31.9°, absent in the compacted loess, indicating the addition of FGD gypsum induces mineralogical changes. These peaks correspond to CaSO_4_·2H_2_O, identified by Jade software. The mineral composition of these peaks, primarily consisting of calcium, sulfur, and oxygen, is consistent with the structure of CaSO_4_·2H_2_O. As the FGD gypsum content increases, the intensity of the peaks at 14.8° and 31.9° also increases, indicating enhanced mechanical properties of the loess. This improvement is attributed to the deposition of CaSO_4_·2H_2_O on the surface of loess particles, where it either forms a coating or acts as a bridge between particle contacts. These formations contribute to the overall strengthening of the loess.

### Morphological attributes

**Figure 4:**
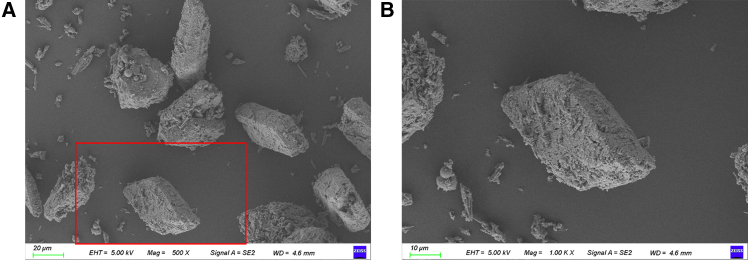
SEM images of loess stabilized with 20% FGD gypsum (A) Image at 500× magnification, with the red square indicating the region selected for further magnification. (B) Corresponding 1000× magnified image of the marked area.

The reaction products produced from FGD gypsum and loess samples with different FGD gypsum contents were examined by SEM. The structure of FGD gypsum exhibits distinct crystalline characteristics critical to its functionality. SEM images at both 500x and 1000x (Figures 4A and 4B) magnifications reveal a well-defined crystalline morphology, with individual particles displaying smooth surfaces interspersed with sharp edges. This crystalline structure indicates a high degree of order, contributing to the mechanical stability of the material. These finer textures may increase the specific surface area, facilitating better bonding and interaction potential.

**Figure 5:**
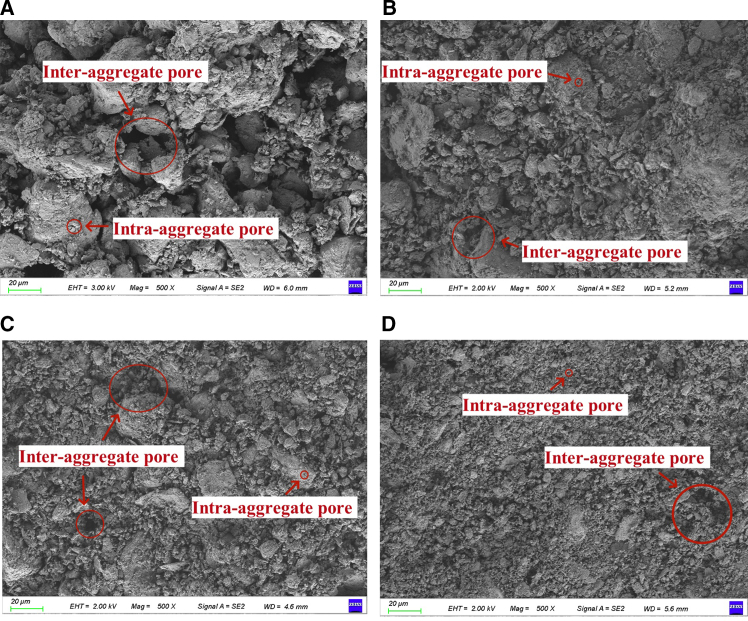
Morphology of samples with different FGD gypsum (A) 0%; (B) 5%; (C) 10%; (D) 20%.

The SEM images (Figure 5) show distinct particle aggregations across the samples, magnified at 500––– for those containing 0%, 5%, 10%, and 20% FGD gypsum (Figure 5A–5D). In the compacted loess sample with 0% FGD gypsum, clay and silt particles tend to agglomerate, where clay particles are either randomly distributed on the surface of silt particles or function as connecting bridges. These clay-silt agglomerates primarily consist of interconnected clay bridges and buttresses,^34^ indicating that the aggregation is a characteristic of the loess structure rather than a result of isolated particle interactions.^35^ Two principal types of pores are identified within loess, i.e., intra-aggregate pores, which are formed within the aggregates, and inter-aggregate pores, situated between the aggregates. This delineates a microstructural domain comprising the aggregates and their internal pore spaces. The void network among the aggregates, recognized as inter-aggregate pores, represents the macrostructure of the material.^36^ The abundance of inter-aggregate pores within compacted loess, combined with numerous point-to-point particle contacts, significantly contributes to the instability of the loess structure, as shown in Figure 5A. Incorporating 5% FGD gypsum (Figure 5B) significantly changes the microstructural characteristics and promotes a more compact arrangement of aggregates, effectively reducing inter-aggregate pore spaces. Additionally, the large lateral surface area of FGD gypsum restricts aggregate movement, enhancing the structural integrity and strength of the samples. This phenomenon is evident in Figure 5C, where the pores between aggregates are noticeably minimized, and the aggregates exhibit tighter packing.

When the FGD gypsum content reached 20%, as shown in Figure 5D, the gypsum particles filled the voids within the aggregate structure, significantly improving the overall strength of the sample. The EDXS results provide further insight into the distribution of FGD gypsum within the composite structure (Table 1). Compared to the original loess, adding FGD gypsum resulted in varying degrees of reduction in the concentrations of elements, such as O, Na, Mg, Al, Si, K, and Fe. Conversely, the levels of Ca and S elements increased, attributable to the primary composition of FGD gypsum as CaSO_4_·2H_2_O. Since the main constituent mineral of loess aggregates is quartz (SiO_2_), the distribution of the Si element can represent the distribution of aggregates. The spatial distribution of the S element, as depicted in EDXS, indicates that the S element is predominantly localized around the aggregates (Figure S3), corroborating the observations made through SEM analysis.

**Table 1:** Chemical composition of samples

Element	O	Na	Mg	Al	Si	S	K	Ca	Fe
Compacted loess	54.52%	1.47%	1.9%	9.36%	19.99%	/	2.99%	5.75%	4.02%
Sample with 20% FGD gypsum	46.85%	0.28%	1.26%	3.36%	7.9%	13.23%	1.24%	23.11%	2.77%

### Changes in 2D microstructural pore characteristics evolution obtained from scanning electron microscopy images

#### Pore area ratio

The Avizo software facilitates enhanced interpretation of SEM results by comparing void fractions and total pore area ratios derived from SEM images.^25^ The pore area ratio (PAR) was calculated by Equation 1.

(Equation 1) $$PAR=\frac{A_{totalpore}}{A_{total}}\times 100\%$$

where *A*_*totalpore*_ is the total pore area and *A*_*total*_ is the area of the SEM image.

The Avizo software analysis of compacted loess samples (without FGD gypsum) yielded a total pore area ratio of 40.15%, closely aligning with the measured porosity of 42.38%. The porosity value was not obtained through direct laboratory measurement but instead calculated using classical soil mechanics theory. Based on the known dry density (1.55 Mg/m^3^) and the specific gravity of soil particles (given in Table S1), the porosity was computed using Equation 2.

(Equation 2) $$n=1-\frac{\rho_{d}}{G_{s}\rho_{w}}$$

where *n* is the porosity (%); *ρ*_*d*_ is the dry density of the soil (Mg/m^3^), *G*_*s*_ is the specific gravity of soil particles, and *ρ*_*w*_ is the density of the water (taken as 1.00 Mg/m^3^).

**Figure 6:**
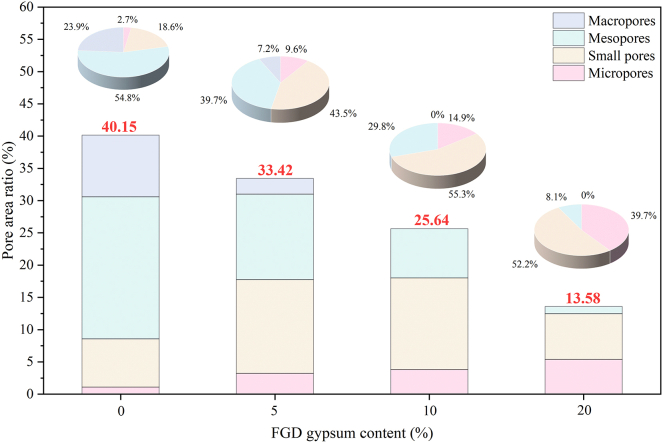
Histogram of the distribution of different pore categories as a percentage of the total pore area

Lei^37^ classified loess pores as micropores (0–2 μm), small pores (2–8 μm), mesopores (8–32 μm), and macropores (>32 μm). Small pores, mesopores, and macropores are predominantly inter-aggregate, while micropores exist primarily within aggregates.^38^ This classification is consistent with the distinctions between intra- and inter-aggregate pores. Figure 6 presents the total area percentages for each pore category alongside the total pore area ratios for samples with varying FGD gypsum content, as analyzed by the Avizo software (Figure 5). The compacted loess (0% FGD gypsum) exhibited a total pore area ratio of 40.15%, which was used as the baseline for comparison. After adding 5%, 10%, and 20% FGD gypsum, the total pore area ratio decreased progressively to 33.42%, 25.64%, and 13.58%, respectively, corresponding to a 16.76%, 36.14%, and 66.18% reduction from the control specimen. Regarding pore category evolution, the macropore area proportion significantly decreased from 23.9% in the control specimen to 7.2% at 5% FGD gypsum, and became almost zero at both 10% and 20% dosages. For mesopores, reductions of 27.66%, 45.68%, and 85.18% were observed at 5%, 10%, and 20% FGD gypsum, respectively, though the small pore fractions increased by 133.90%, 197.88%, and 180.79% for the same FGD gypsum contents. A notable rise in micropores was also observed, consistent with the EDXS results, elucidating that FGD gypsum addition fills inter-aggregate pores, which transforms macropores and mesopores into smaller pores and micropores, densifying the pore structure.

#### Pore shape factor

In this study, roundness was introduced to quantify the pore shape distribution in samples with different FGD gypsum content, as given in Equation 3.

(Equation 3) $$Roundness=\frac{4\pi A_{pore}}{P_{pore}^{2}}$$

where *A*_*pore*_ and *P*_*pore*_ are the area and perimeter of each single pore, respectively.

**Figure 7:**
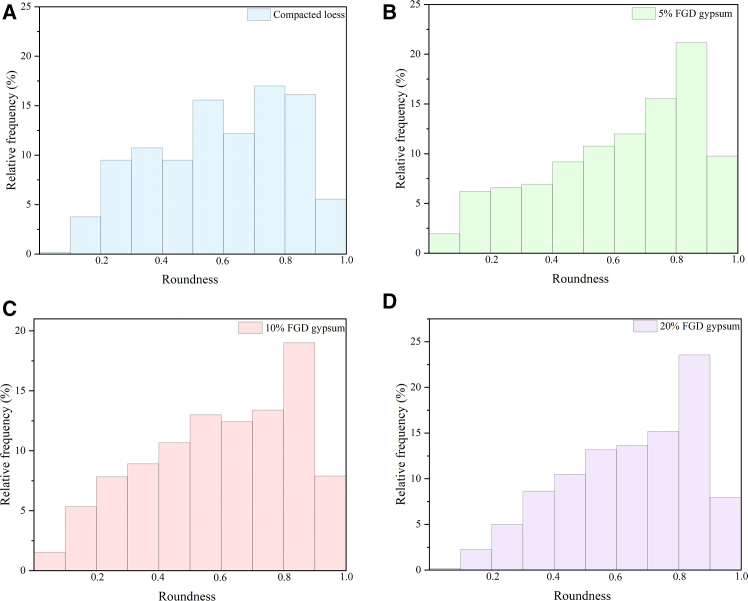
Variation in the pore shape factor

Roundness values range from 0 to 1, with values closer to 1 indicating a pore shape that more closely approximates a circle. The distribution of roundness for samples with varying FGD gypsum content is shown in Figure 7. Roundness measurements were categorized into ten intervals ranging from 0.0 to 1.0, with increments of 0.1. All samples exhibited similar overall distributions, with the peak frequency consistently appearing in the 0.8–0.9 range. However, a progressive increase in the proportion of highly rounded pores was observed with increasing FGD gypsum content. In particular, the proportion of pores falling within the 0.8–0.9 roundness interval increased from 16.10% in the compacted loess (0% FGD gypsum) to 21.15%, 19.01%, and 23.54% at FGD gypsum dosages of 5%, 10%, and 20%, respectively, i.e., an increase of 31.4%, 18.1%, and 46.2%, compared to the control group. This increase may be attributed to the increasing number of small pores and micropores with FGD gypsum content. Yuan et al.^20^ confirmed that the shape of micropores and small pores is predominantly circular, explaining the observed roundness distribution.

#### Pore angle distribution

Pore angle is defined by the spatial orientation of pores, determined by the alignment between the long axis of the pore and the x axis.^39^ Eighteen specimen groups were measured, each with a 10° interval for pore orientation angles. The statistical results of these measurements were visualized through rose diagrams (Figure S4). For compacted loess, the distribution of pore angles is notably concentrated, with the 80°–90° range accounting for 15.67% of the total pore orientations. However, when FGD gypsum is added to the loess, the concentration of pore angles significantly decreases. The angle distribution lacks a distinct peak in samples containing 20% FGD gypsum. Additionally, adding FGD gypsum tends to shift pore orientation toward more horizontal angles, indicating a more dispersed pore arrangement.

### Changes in 3D microstructural pore characteristics evolution obtained from CT images

**Figure 8:**
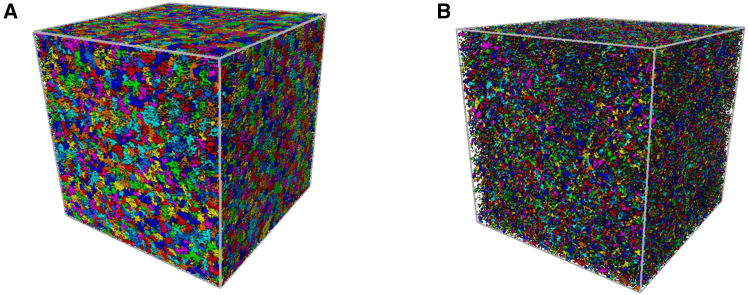
3D structures (A) Compacted loess and (B) sample with 20% FGD gypsum content.

In this study, based on the results of the 2D analysis, the CT images of compacted loess and the sample with 20% FGD gypsum content (as shown in Figure 8) were also analyzed using Avizo software to establish their 3D structures and extract 3D microstructural pore parameters. The pore volume fractions of compacted loess and 20% FGD gypsum samples were 39.71% and 13.22%, respectively, consistent with the SEM observations, corroborating the reliability of the Avizo-based analysis. The 20% FGD gypsum content was selected for 3D imaging because it exhibited the highest UCS and the most pronounced microstructural improvement within the tested dosage range (0–20%). This allowed for a clearer investigation of the micro-macro linkage between mechanical enhancement and pore structure evolution.

**Figure 9:**
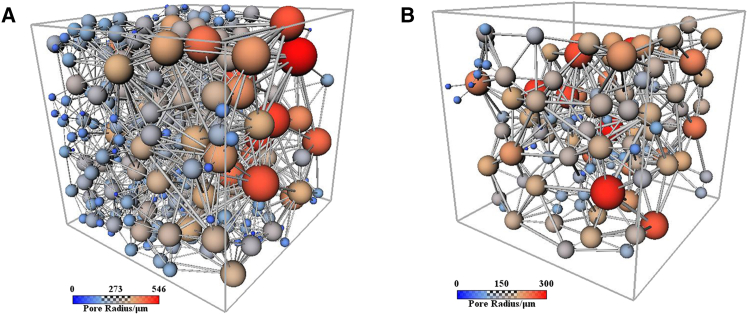
3D pore network model (A) Compacted loess, (B) sample with 20% FGD gypsum content. Spheres represent the pores, and the sticks represent the pore throats interconnecting them.

A 3D pore structure was reconstructed using Avizo software. The 3D pore network model (PNM), representing a network of interconnected pores and throats, is widely accepted for evaluating key macroscopic transport properties, such as capillary pressure and residual saturation.^40^ The pore space was segmented into distinct pore bodies using a specialized algorithm, with the contact area between adjacent pore bodies defining the pore throats. A pore body represents a larger cavity, while the pore throat refers to the narrower passage connecting these cavities (Figure 9). The PNM was generated based on pore segmentation. In this model, nodes signify the intersections of neighboring apertures, corresponding to pore bodies, interconnected by bonds representing the pore throats. In the compacted loess sample (Figure 9A), the pore structure displays a greater number of larger pore bodies (represented by the red and orange spheres), with a relatively high variation in pore size. This indicates a more open pore network, which could lead to lower overall strength due to larger voids. The wider pore throats suggest potential pathways for fluid movement, but the irregular distribution may result in inconsistent mechanical behavior under stress. In contrast, the loess sample with 20% additive (Figure 9B) shows a much denser and more uniform pore network. The pore bodies are significantly smaller, with most radii clustering around the lower end of the spectrum. This reduction in pore size and overall porosity suggests an increase in compactness, which correlates with enhanced mechanical strength.

#### EqD distribution of pores from CT images

**Figure 10:**
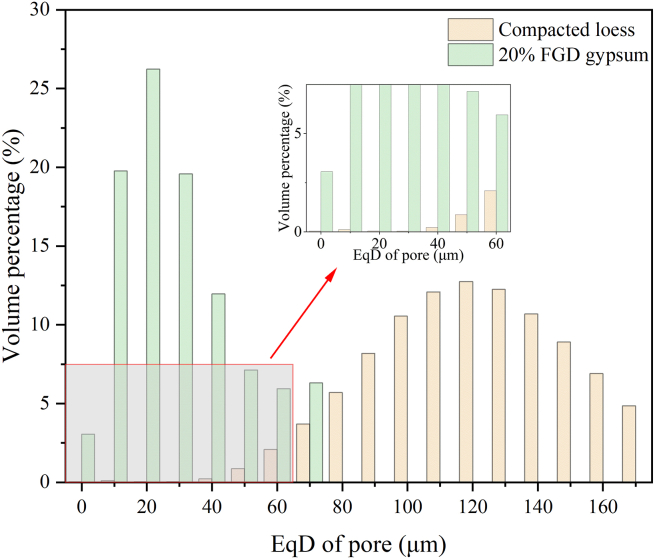
EqD distribution of pores from CT images

The 3D pore sizes were characterized by EqD, and the corresponding results are shown in Figure 10. The pore volume occupation ratio of both compacted loess and 20% FGD gypsum samples showed an increasing and then decreasing trend with the increase of pore size. However, the maximum size of pores in the 20% FGD gypsum samples was less than 80 μm compared to the compacted loess. Therefore, it was demonstrated that adding FGD gypsum significantly reduced the pore size between the sample structures. The pore structure characterization using Avizo software presents clear disparities in the pore diameters between 2D and 3D models, with the 3D results consistently showing larger pore sizes, attributed to several inherent factors in the dimensional analysis of porous media. In a 2D analysis, the pore size is measured based on cross-sectional views of the sample, where only the visible portion of the pores is captured. This can result in underestimating the actual pore size because pores extending in the third dimension (out of the plane of the 2D section) are truncated. Essentially, the 2D analysis provides a “slice” of the full pore geometry, capturing only a part of the pore volume, especially in irregularly shaped or elongated pores. Consequently, this method often leads to smaller pore diameters, as it misses the full extent of the pore space.

In a 3D analysis, Avizo reconstructs the entire pore network, capturing the full geometry and connectivity of the pores in all three spatial dimensions. This comprehensive reconstruction allows a more accurate representation of pore volumes and diameters. 3D analysis can also capture their full size, including their depth, by considering the true spatial extent of the pores, which is not visible in 2D projections. Thus, 3D models typically report larger pore diameters due to this holistic representation of the pore space. Although there is a difference between the 2D and 3D results, the pore size results are not orders of magnitude different, so both can support quantitative analysis of the sample microstructure.

#### Shape factors of pores from CT images

In this study, three parameters, i.e., flatness, elongation, and sphericity, were introduced to analyze the 3D pore shape factor, as given in Equations 4, 5, and 6

(Equation 4) Flatness=T/B

(Equation 5) Elongation=1−B/L

(Equation 6) $$Sphericity={\left(A^{3}/\left(36\times \pi \times V_{2}\right)\right)}^{1/3}$$

The flatness was quantified by the thickness-to-width ratio, with values ranging from 0 to 1, where a higher value indicates a flatter shape. Here, *T* and *B* denote the thickness and breadth of the object, respectively. Elongation was measured by the ratio of the difference between the length and breadth to the length, also with values between 0 and 1, where a larger value suggests a more elongated shape; *L* represents the object length. Sphericity assesses how closely the object shape resembles a perfect sphere, with values ranging from 0 to 1, where a higher value signifies greater sphericity; *A* and *V* denote the area and volume of the object, respectively. The mean values of the 3D pore shape factor results for compacted loess and 20% FGD gypsum samples are presented in Table 2. Sphericity showed an increasing trend with FGD gypsum addition, whereas flatness showed the opposite result. Elongation variation with the addition of FGD gypsum was minor.

**Table 2:** Shape factors of pores

	Compacted loess	20% FGD gypsum sample
Flatness	0.5774	0.5532
Elongation	0.4948	0.4944
Sphericity	0.6733	0.6932

#### Orientation angle of pores from CT images

The orientation of pores in 3D space was characterized using the maximum Feret diameter direction,^25^ as shown in Figure S5A. In this context, φ denotes the angle between the pore and the z axis, consistent with the maximum principal stress direction, ranging from 0° to 90°. Meanwhile, θ represents the orientation of the pore projected onto the XY-plane, varying between −180° and 180°. The pore orientation angle pairs (φ, θ) were classified into 31 discrete groups, as shown in Figure S5B. The distribution of pore volume percentages of compacted loess and loess incorporated with 20% FGD gypsum in these groups is shown in Figure S5B. The compacted loess exhibited distinct peaks in the ranges of (47°, 55°), (72°, 69°), (75°, 41°), and (85°, 124°), whereas the 20% FGD gypsum samples have a more uniform angle distribution.

### Mechanisms for flue gas desulfurization gypsum-stabilized loess

**Figure 11:**
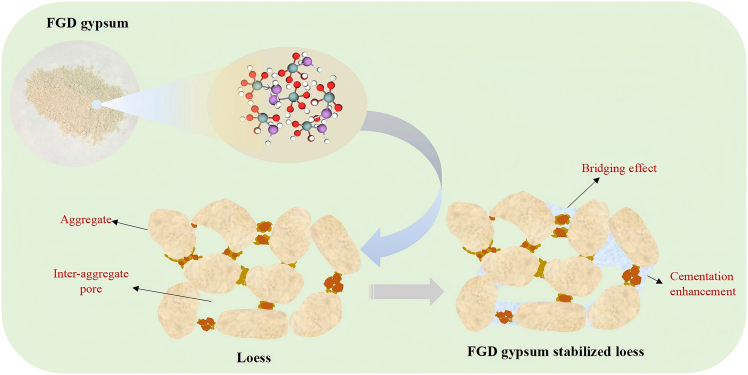
Mechanism of FGD gypsum stabilized loess

The significant increase in the UCS of loess stabilized with FGD gypsum can be explained by microstructural changes, including enhanced cementation, modifications in mineral composition, and pore structure optimization. A schematic diagram of FGD gypsum stabilization is shown in Figure 11. The underlying mechanisms of the role of FGD gypsum in loess stabilization were revealed through FTIR, XRD, SEM, and CT scanning. FTIR analysis showed the emergence of new absorption peaks in the loess samples after the addition of FGD gypsum, particularly at 1624 cm^−1^ and 1155 cm^−1^, corresponding to hydroxyl (–OH) and sulfate (–SO_4_) groups, respectively. These functional groups could enhance the chemical bonding between loess particles, increasing interparticle cohesion and thereby improving the overall structural stability of the loess, which led to a significant increase in UCS. XRD analysis further revealed changes in the mineral composition of the loess following the introduction of FGD gypsum, particularly the formation of CaSO_4_·2H_2_O, which played a critical role. As the FGD gypsum content increased, the intensity of the characteristic peaks of CaSO_4_·2H_2_O in the XRD spectra also increased. This CaSO_4_ deposited between loess particles, creating a “bridging” effect that strengthened the physical bonding between particles, significantly enhancing the mechanical strength of the loess. These changes in mineral composition were one of the key factors driving the improved mechanical properties of the stabilized loess. Morphological analysis using SEM and CT scanning demonstrated the significant impact of FGD gypsum on the pore structure of the loess. SEM images revealed that adding FGD gypsum filled large inter-aggregate pores, reducing the overall pore volume and increasing the compactness of the loess. This phenomenon was further confirmed through 3D pore structure reconstruction via CT scanning. As the FGD gypsum content increased, the pore structure became denser and more uniform, significantly reducing porosity. This more compact pore structure improved the load-bearing capacity of the material, reduced stress concentration, and significantly enhanced the UCS.

Quantitative analysis of the SEM and CT results further clarified the contribution of pore structure optimization to the increase in UCS. FGD gypsum addition reduced the number of large pores and increased the proportion of small and micropores, which became more rounded and uniform. As the FGD gypsum content increased, the uniformity of the pore network improved, and the pore shapes became more regular, effectively mitigating failure caused by stress concentration. This process contributed to the significant improvement in compression strength, further validating the crucial role of pore structure optimization in enhancing the strength of the stabilized loess.

Overall, adding FGD gypsum significantly improved the UCS of loess by enhancing cementation between particles, altering mineral composition, and optimizing the pore structure. The synergistic effects of increased cementation, mineral deposition, and pore structure optimization during stabilization resulted in a denser and stronger material. These insights into the microstructural mechanisms provide important theoretical support for further developing and applying FGD gypsum for soil stabilization.

### Environmental performance

**Figure 12:**
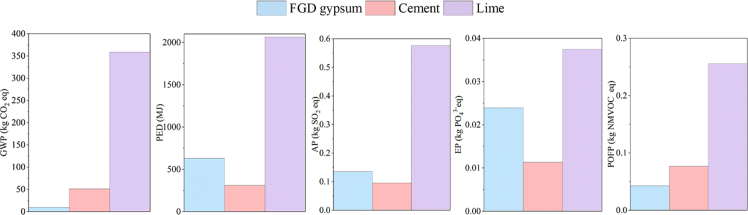
Environmental impacts of the 1 m^3^ loess reinforcement by stabilizers

Figure 12 shows the environmental impacts of commonly used stabilizers for reinforcing 1 m^3^ of loess under conditions where the compression strength is comparable to that of silica fume-stabilized loess. Global Warming Potential (GWP) is particularly significant due to its direct link to greenhouse gas emissions and its critical role in global sustainability goals. FGD gypsum demonstrates a remarkably low GWP of approximately 10 kg CO_2_-eq, significantly lower than cement (∼51 kg CO_2_-eq) and lime (∼359 kg CO_2_-eq), i.e., a reduction of about 80% and 97% in CO_2_ emissions compared to lime and cement, respectively, per m^3^ of stabilized loess. The substantial reduction is primarily attributed to the absence of the energy-intensive calcination process required for lime and cement production, a major contributor to CO_2_ emissions.

In terms of photochemical ozone creation potential (POCP), FGD gypsum also exhibits superior performance, with an impact of approximately 0.04 kg NMVOC-eq, compared to 0.08 kg NMVOC-eq for cement and 0.26 kg NMVOC-eq for lime. In addition, its performance in primary energy demand (PED), acidification potential (AP), and eutrophication potential (EP) categories is moderate, whereas FGD gypsum remains competitive with cement and lime, indicating its overall environmental viability. These findings underscore the environmental superiority of FGD gypsum as a stabilizer for loess, particularly in reducing greenhouse gas emissions. Substantive progress can be made toward decarbonization goals by replacing traditional high-emission stabilizers with FGD gypsum, reinforcing its potential as a sustainable and climate-friendly alternative for soil stabilization applications.

### Conclusions

In this study, an integrated evaluation of the mechanical, microstructural, and environmental performance of loess stabilized with flue gas desulfurization (FGD) gypsum was conducted, revealing its potential as a low-carbon alternative to conventional binders. The main conclusions drawn from the obtained results are as follows:

• The UCS of loess significantly increased with FGD gypsum content. A dosage of 20% could help achieve a UCS of 374.3 kPa, i.e., a 398% improvement over untreated loess, signifying its strong cementation capacity and engineering viability.
• Microstructural analyses (FTIR, XRD, SEM, and CT) revealed critical mechanisms, including improved cementation, enhanced mineral deposition (CaSO_4_·2H_2_O formation), and optimized pore structure. Quantitative image processing confirmed a denser and more uniform pore network, with reduced porosity and increased small and micropores, directly contributing to improved mechanical properties.
• Life cycle assessment showed that FGD gypsum stabilization drastically reduced the global warming potential to ∼10 kg CO_2_-eq/m^3^—an ∼80% and ∼97% decrease compared to cement and lime, respectively, elucidating its ecological advantages.

These results establish a clear micro-macro strengthening mechanism and reinforce the suitability of FGD gypsum for sustainable soil improvement in loess regions.

### Limitations of the study

This study investigated the stabilizing effects of FGD gypsum on loess within a dosage range of up to 20%. Although a continued increase in UCS was observed with higher dosages in this range, the behavior of loess at FGD gypsum contents exceeding 20% remains unexplored. It is, therefore, uncertain whether further strength gain, performance improvement, or even detrimental effects might occur beyond this dosage. Also, the conclusions are based on laboratory-scale tests under controlled conditions. Field-scale validation and broader dosage studies are necessary to confirm the optimal application range and practical feasibility of FGD gypsum stabilization in geotechnical engineering projects.

## Resource availability

### Lead contact

Requests for further information and resources should be directed to and will be fulfilled by the lead contact, Kangze Yuan (kangze_yuan@nwu.edu.cn).

### Materials availability

This study did not generate new unique reagents.

### Data and code availability

• All data reported in this article will be shared by the lead contact upon request.
• This article does not report the original code.
• Any additional information required to reanalyze the data reported in this article is available from the lead contact upon request.

## Acknowledgments

This study was substantially supported by the 10.13039/501100002858China Postdoctoral Science Foundation (Grant no. 2024MD753992), the Shaanxi Geotechnical Mechanics and Engineering Young Talent Support Program Project (Grant no. YESS2024005), the general projects of the 10.13039/501100017596Shaanxi Province Natural Science Basic Research Program (2025JC-YBQN-435), the 10.13039/100014718National Natural Science Foundation of China (Grant No. 42372320, Grant No. 41972292), the Innovation Capability Support Program of Shaanxi Province (Grant No. 2021TD-54), and the 10.13039/501100015401Key Research and Development Projects of Shaanxi Province (Grant No. 2022ZDLSF06-03).

## Author contributions

Writing – original draft, K.Y.; writing - review and editing, W.X.; conceptualization, K.Y. and W.X.; methodology, K.Y. and W.X.; formal analysis, K.Y.; supervision, W.X.; investigation, K.Y., W.X., X.GH., Q.L., and X.L.

## Declaration of interests

The authors declare no competing interests.

## STAR★Methods

### Key resources table

**Table:** 

REAGENT or RESOURCE	SOURCE	IDENTIFIER
**Software and algorithms**		

Thermo Scientific™ Amira-Avizo Software for 3D Visualization and Analysis	Northwest University	https://www.thermofisher.com/sg/en/home

**Other**		

Nicolet iS50 spectrometer	Thermo Fisher Scientific, USA	https://www.thermofisher.com/order/catalog/product/912A0760
Bruker AXS X-ray diffractometer (D8 Advance)	Bruker	https://www.directindustry.com/prod/bruker-axs-gmbh/product-30028-1821023.html
Zeiss Sigma 500 microscope	Zeiss, Germany	https://emin.asia/zeisssigma-500-zeiss-sigma-500-electron-microscopy-133987/pr.html
ZEISS Xradia 520 Versa X-ray micro-CT system	Zeiss, Germany	https://www.zeiss.com/microscopy/en/products/x-ray-microscopy/versaxrm.html

### Method details

#### Raw materials

The study area is in Yulin City, located in the northern region of the Loess Plateau. Yulin is a major urban center characterized by high population density and limited expanses of flat terrain. The area has a temperate continental monsoon climate, distinguished by four distinct seasons, significant diurnal temperature variations.^41^ According to the Yulin Statistical Yearbook 2023, the mean annual temperature in Yulin was approximately 9.6 °C, and the total precipitation reached 490 mm. The soil samples comprised Q_3_ loess, referred to as Malan loess in China, collected from depths ranging between 5.0 m and 5.5 m below the surface. These were cast into cylindrical specimens (0.1 m diameter and 0.2 m height), sealed in paraffin-coated sample buckets, and packed in straw-filled wooden crates to prevent disturbance during transport.^17^ Upon arrival at the laboratory, the physical properties of the loess were analyzed following ASTM standards,^42^ with key results summarized in Table S1. As shown in Figure S1A, the particle size distribution reveals that the loess primarily comprises silt (71.49%), with lesser fractions of sand (25.96%) and clay (2.55%).^43^ The FGD gypsum was sourced from a coal-fired power plant in Yulin City (Figure S1B). It is a fine, white to pale yellow powder, primarily composed of calcium sulfate dihydrate (CaSO_4_·2H_2_O), with no detectable odor. The particle size distribution of FGD gypsum was analyzed using laser diffraction (Figure S1A). Results showed that particles smaller than 2 μm accounted for 6.24%, those between 2 and 75 μm made up 80.85%, and particles larger than 75 μm constituted 12.91%, indicating a predominantly fine-grained composition favorable for pozzolanic reactions and pore filling.

#### Sample preparation

The loess and FGD gypsum were crushed, sieved through a 0.075 mm mesh, and then dried at 105°C for 8 hours to remove water. The loess was subsequently mixed with varying mass percentages of FGD gypsum to evaluate its effect on the properties of the loess. Three samples with 5%, 10% and 20% FGD gypsum content were prepared. The water content was then adjusted to 10% (natural water content) by adding distilled water to the dried loess-FGD gypsum mixture. Finally, the samples were placed in polythene bags and humidors for 2 days to ensure uniform moisture distribution. In order to better explain the effect of FGD gypsum on the changes in the properties of loess, a control experiment was also carried out on loess without FGD gypsum addition. Since dry density is the main control parameter affecting the construction quality of compacted loess, the average dry density of compacted soils at the construction site in the study area ($\rho_{d}$=1.55 Mg/m^3^) was used as the target value for sample preparation.^44^ Therefore, all samples were statically compacted by a triaxial compactor into the corresponding test moulds at the target dry density of 1.55 Mg/m^3^. After compaction, all specimens were cured under identical conditions at 22 °C and 80% RH for 7 days before unconfined compression strength testing, ensuring consistency across the dataset.

#### Test procedure

Various experimental tests were conducted, including mechanical strength testing and multiscale microstructural characterization, to systematically evaluate the effects of FGD gypsum on loess. The test procedures are described as follows.

##### Unconfined compression strength (UCS) test

Unconfined compression tests were performed following ASTM D2166M-2016^45^ to assess the mechanical performance of loess stabilized with various contents of FGD gypsum. Each sample group (0%, 5%, 10%, 20% FGD gypsum) consisted of three specimens. The UCS values were recorded, and the average value was reported.

##### Fourier transform infrared (FTIR) spectroscopy

FTIR analysis was performed to detect changes in functional groups associated with mineralogical transformations after adding FGD gypsum. It is a vibrational spectroscopic technique used to identify molecular functional groups by measuring the absorption of infrared radiation as it interacts with the chemical bonds within a sample. When infrared light passes through a sample, specific wavelengths are absorbed depending on the types of bonds present (e.g., O–H, C=O, S=O), producing a spectrum that acts as a “fingerprint” of the chemical composition of the material. The spectra were recorded using a Thermoelectric Nicolet iS51 spectrometer in the range of 4000–400 cm^-1^ using the KBr pellet technique. Equal sample amounts were used for each measurement to ensure comparability. Characteristic peaks corresponding to sulfate groups, hydroxyls, and silicates were analyzed to identify the formation of different compounds, such as gypsum or ettringite.

##### X-Ray diffraction (XRD)

XRD analysis was used to identify mineralogical compositions and newly formed crystalline phases. The measurements were conducted using a Bruker D8 Advance diffractometer with Cu Kα radiation (λ = 1.5406 Å), operating at 40 kV and 30 mA, and scanning across a 2θ range of 5°–90°. Samples were oven-dried and ground to pass through a 75 μm sieve before testing.

##### Scanning electron microscopy (SEM)

SEM was employed to examine morphological changes and cementation structures at the microscale. Rectangular specimens (2 cm × 1 cm × 1 cm) were cut from the cured samples. After air-drying, these samples were broken by hand 1–2 h before the observations to obtain a fresh fractured surface. SEM images were obtained using a Zeiss Sigma 500 microscope after gold coating on the fractured surface. Energy-dispersive X-ray spectroscopy (EDXS) was also applied to specific points to determine elemental compositions related to FGD gypsum hydration products.

##### X-Ray computed tomography (CT)

Micro-CT scanning was conducted on cubic samples (approximately 5 mm^3^) extracted from the central region of each specimen to capture the three-dimensional pore network characteristics of compacted loess. After undergoing the same pre-treatment procedures as for SEM observations, the samples were scanned using a ZEISS Xradia 520 Versa X-ray micro-CT system. The resulting grayscale image stacks were processed and analyzed using Avizo software,^25^ enabling the reconstruction and quantification of key microstructural parameters. This 3D characterization provided a complementary perspective to the 2D pore area data obtained via SEM, allowing for a more comprehensive and quantitative assessment of the pore network evolution induced by FGD gypsum stabilization.

#### Life cycle assessment (LCA)

##### Goal and scope definition

LCA was performed in this study in compliance with ISO 14040:2006^46^ and ISO 14044:2006^47^ standards to evaluate the environmental impacts of FGD gypsum-stabilized loess compared to cement- and lime-stabilized loess. The functional unit was defined as the materials and energy required to stabilize 1 m^3^ of loess. Key environmental hotspots were identified by assessing the impacts of producing conventional stabilizers (cement and lime) and comparing them to FGD gypsum. The analysis highlights the excellent environmental performance of FGD gypsum, signifying its potential to mitigate sustainability challenges and promote its adoption for loess stabilization.

A cradle-to-grave approach of LCA was performed, encompassing the production of stabilizers (cement, lime, and FGD gypsum) and loess stabilization processes, as illustrated in Figure S2. Energy consumption during site preparation, material mixing, and soil compaction was excluded from the analysis due to insufficient data. Similarly, the use-phase and end-of-life stages were omitted as they have negligible environmental impacts. This system boundary approach ensures a focused assessment of the environmental implications of stabilizer production and application processes.

##### Life cycle inventory analysis

The inventory analysis forms the backbone of this assessment, systematically identifying all inputs and outputs across the stabilization process. Within the defined system boundaries, inventory data were gathered from relevant literature. Calculations were performed using the eFootprint platform, with datasets from the Ecoinvent v3.1 database and the CLCD.^48^ The assessment assumed a global average electricity mix as the energy supply for production processes, ensuring consistency in the input parameters. Results from previous studies^49^^,^^50^ revealed that loess stabilized with 20% FGD gypsum achieved UCS values comparable to samples stabilized with 16% lime (7-day curing) or 4% cement (7-day curing). These findings underscore the efficacy of FGD gypsum as a stabilizer, offering comparable performance to conventional materials under similar conditions. The life cycle inventory (LCI) of materials required to stabilize 1 m^3^ of loess using different stabilizers is detailed in Table S2. This inventory provides critical insights into the material and energy demands associated with each stabilizer type, forming the foundation for evaluating their environmental and economic implications.

##### Life cycle impact assessment

An appropriate environmental impact category must be selected per the evaluation methodology, as suggested by Ferrao.^51^ This study assessed ecological loads and effects using intermediate indicators derived from these loads. To ensure consistency with international standards, the study adopted the method outlined in the “operational guide to the ISO standards” published by the Center of Environmental Science at Leiden University (CML) in 2001. The selected impact categories include Global Warming Potential (GWP), Acidification Potential (AP), Eutrophication Potential (EP), and Photochemical Ozone Creation Potential (POCP). Primary Energy Demand (PED) was incorporated based on the Cumulative Energy Demand (CED) approach, providing a comprehensive assessment of energy usage across the system.

### Quantification and statistical analysis

This study primarily focuses on the mechanical properties, mineralogical compositions, and microstructural characteristics of loess stabilized with FGD gypsum. Quantitative data, including unconfined compressive strength (UCS), pore structure parameters, and elemental distributions, were obtained through laboratory experiments and image analyses. Data processing and plotting were conducted using OriginPro 2023 and Microsoft Excel 2019. Each experimental value (e.g., UCS results) represents the average of at least three parallel specimens (n = 3), unless otherwise stated. For microstructural analyses (e.g., SEM and CT-based 3D reconstruction), representative images were selected. No hypothesis testing or statistical comparison between groups was performed. Therefore, standard statistical tests (e.g., t-test or ANOVA) and error bars are not included in most figures. All relevant quantitative^50^^,^^51^ data and analysis details are provided in the corresponding figure legends and results and discussion section.
