# Supplementary material for: From waste to strength: The role of FGD gypsum in loess stabilization and its environmental benefits
Source: iScience. 2025 Oct 30;28(11):113909. doi: 10.1016/j.isci.2025.113909 (PMC12666362; doi:10.1016/j.isci.2025.113909)
Supplement: Document S1. Figures S1–S5 and Tables S1–S2 [file mmc1.pdf]

iScience, Volume *28*

Supplemental information

From waste to strength: The role of FGD gypsum in loess stabilization and its environmental benefits

Yuan Kangze, Xie Wanli, Gao Xuanyu, Li Xinyu, and Liu Qiqi

Supplemental Figures

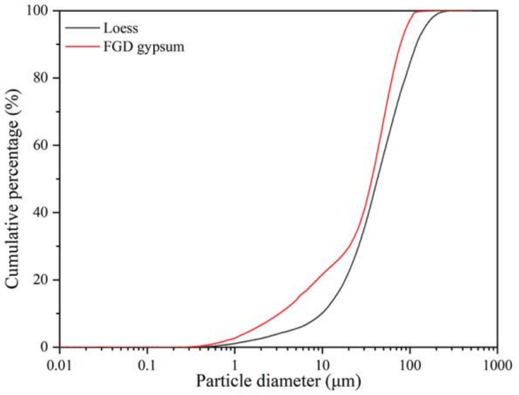

(a)

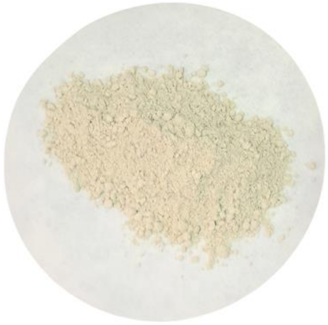
(b)

Fig. S1. Particle size distribution and appearance of loess and FGD gypsum.

a: Particle size distributions of original loess and FGD gypsum.

b: Photograph of FGD gypsum particles.

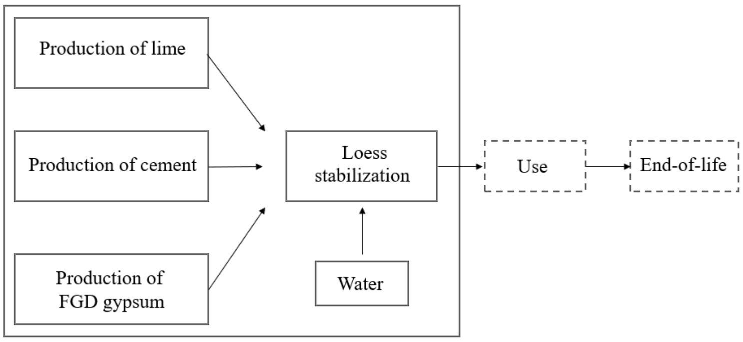

Fig. S2. System boundary used in environmental analysis.

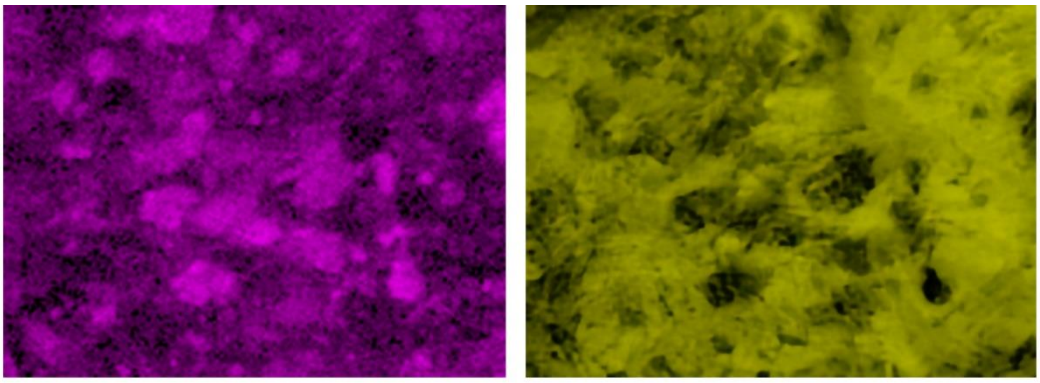

(a) (b)

Fig. S3. EDX element mapping of loess stabilized with 20% FGD gypsum.

a: Si element.

b: S element.

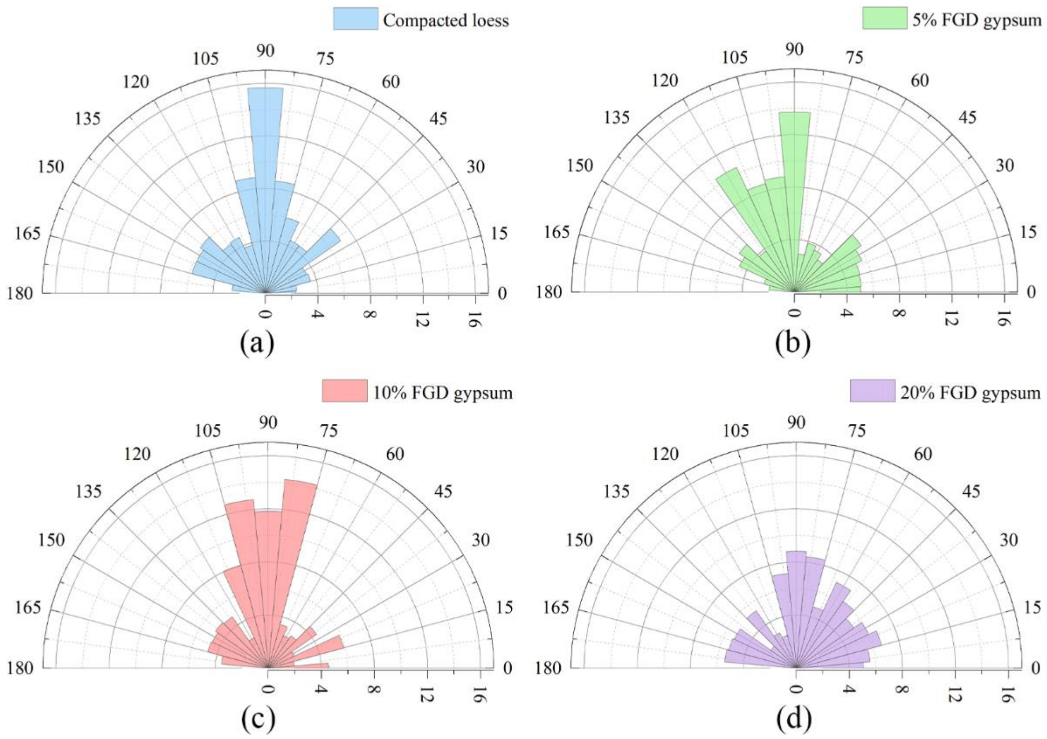

Fig. S4. Distribution of pore angles of loess samples.

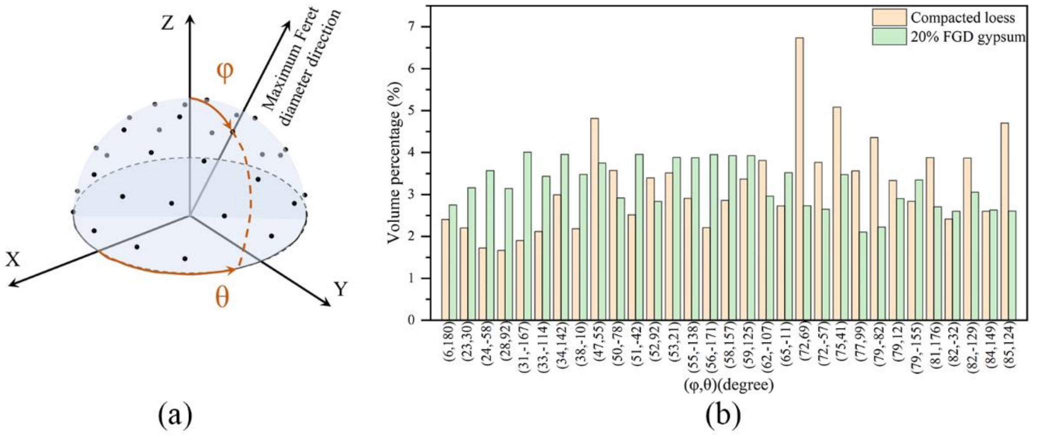

Fig. S5. Classification and visualization of pore orientation angles.

a: Schematic representation of the 31 groups of pore orientation angles (modified from Yuan and Fan, [27]).

b: Distribution of pore orientation angles based on the classified groups.

Supplemental Tables

Table S1. Basic physical properties of original loess.

Physical parameter	Value
Clay content (%)	2.55
Silt content (%)	71.49
Sand content (%)	25.96
In situ dry density (Mg/m^3)	1.55
Natural water content (%)	10
Specific gravity	2.69
Plastic limit (w_P/%)	12.8
Liquid limit (w_L/%)	23.6
Plastic index	10.8
Maximum dry density (Mg/m^3)	1.72
Optimum water content (%)	13.1

Table S2. Life cycle inventory of materials used to stabilize 1 m^3 loess.

	Lime	Cement	FGD gypsum	Unit
Lime	265.6			kg
Cement		69.2		kg
FGD gypsum			310	kg
Water	298.3	213.85	155	kg
